# Skin Cleansing without or with Compromise: Soaps and Syndets

**DOI:** 10.3390/molecules27062010

**Published:** 2022-03-21

**Authors:** Dalibor Mijaljica, Fabrizio Spada, Ian P. Harrison

**Affiliations:** Department of Scientific Affairs, Ego Pharmaceuticals Pty Ltd., 21–31 Malcolm Road, Braeside, VIC 3195, Australia; dalibor.mijaljica@egopharm.com (D.M.); fabrizio.spada@egopharm.com (F.S.)

**Keywords:** capacity, charge, ingredient, mildness, pH, cleanser, skin, soap, surfactant, syndet

## Abstract

Products designed to cleanse the skin commonly do so through surfactant action, which leads to the lowering of the surface tension of the skin to facilitate the removal of dirt from its surface. Skin cleansers generally come in one of two types: soap-based and synthetic detergents, or syndets. While the latter can effectively maintain the native skin structure, function and integrity, the former tends to negatively affect the skin by causing barrier disruption, lipid dissolution and pH alteration. Despite this, soap is still often preferred, possibly due to the negative connotations around anything that is not perceived as ‘natural’. It is, therefore, important that the science behind cleansers, especially those designed for the maintenance of healthy skin and the management of common skin conditions such as eczema, be understood by both formulators and end-users. Here, we carefully weigh the advantages and disadvantages of the different types of surfactant—the key ingredient(s) in skin cleansers—and provide insight into surfactants’ physicochemical properties, biological activity and potential effects. Fine-tuning of the complex characteristics of surfactants can successfully lead to an ‘optimal’ skin cleanser that can simultaneously be milder in nature, highly effective and beneficial, and offer minimal skin interference and environmental impact.

## 1. Introduction

The stratum corneum (SC) is the uppermost layer of the skin’s epidermis, and is a biochemically complex but highly organised interface with the external environment. The SC is usually described as a ‘bricks and mortar’ structure in which the protein enriched, flattened corneocytes (dead keratinocytes lacking vital cellular organelles) are the ‘bricks’, and the lipid-rich matrix in which they are embedded is the ‘mortar’ [1]. This lipid matrix predominantly contains ceramides (40–50%), cholesterol (25%) and free fatty acids (10–15%) [2,3,4,5]. These three major classes of lipids within the SC are biophysically and biochemically distinct from other conventional eukaryotic membrane constituents (e.g., glycerolipids, sphingolipids, sterols) that are involved in the structural and functional landscape of the cell membrane’s lipid bilayers [6]. SC lipid precursors are generated during keratinocyte differentiation, and are afterwards intracellularly packaged into lamellar bodies in a highly ordered manner and subsequently enzymatically converted and eventually secreted into the extracellular domain of the SC [2,3,7]. The SC lipids spontaneously and in an orderly manner form multiple bilayers, which interact with corneocyte envelope-bound lipids [4,8]. In deeper layers of the SC, lipids are predominantly ordered into a densely packed orthorhombic crystalline configuration, which acts as a restrictive barrier to liquid transport. Closer to the SC surface, the lipids form a dispersed hexagonal lattice configuration that permits more liquid to pass through more freely [9,10].

The highly ordered SC lipid configuration is characterised by its unique properties, composition, and the specialised compartmentalisation within the lamellar lipid membrane, which maintains SC water content, regulates water flux, and modifies the rate and magnitude of transepidermal water loss (TEWL). Such mechanisms are highly dynamic and function continuously to maintain homeostasis of the SC, the epidermis and the skin in its entirety [2]. As such, the SC is a robust factory that is in operation at all times, ensuring the maintenance of the skin’s adaptive and protective barrier [2,11]. Exogenous stressors can impede the skin’s protective properties, so reducing exposure to, and minimising practices such as skin cleansing with, harsh cleansers (e.g., alkaline soaps) that can cause perturbation and damage to the SC proteins, lipids and natural moisturising factor (NMF) components (e.g., free amino acids, sugars) is critical to decreasing the stress load on the skin’s structural and functional integrity [2].

It is generally recognised [7,9,12,13,14] that all types of skin, from healthy to diseased, infant to aged, need to be kept clean in order to preserve their barrier properties. The main objective of skin cleansing is to remove impurities from the skin’s surface [15,16] and to control its odour, without removing protective SC surface proteins and lipids, affecting skin microbiota [17] or altering pH [13,18]. This can be a substantial challenge, as the skin’s composition and barrier integrity are intricate, inconsistent, and dynamic, as described above [13,18].

As most of the impurities and contaminants that are found on the skin’s surface are not water-soluble, cleansing the skin with water alone is inadequate; hence, there is a need for surfactant-containing products [15]. Unlike water, surfactant-containing products are capable of breaking down and emulsifying most of those skin impurities and contaminants into finer particles and enabling their subsequent detachment and removal from the surface of the skin [3,7]. This makes surfactants a key component of skin maintenance. However, not all surfactants are created equal, with some, namely soap-based surfactants, creating more problems than they address [3,9,10,13,16,17,19,20,21,22,23,24,25,26,27,28,29,30,31].

This review will: (1) compare and contrast the key attributes of soaps and syndets; (2) discuss the advantages and disadvantages of different types of surfactant and provide insight into their physicochemical properties, biological activity and potential effects; (3) provide insight into the skin cleansers that are best suited for particular skin conditions such as eczema; and, (4) elucidate how a deeper understanding of the complex characteristics of surfactant chemistry and its effects on the skin can successfully lead to the development of a new generation of skin cleansers with optimal properties, characterised by mildness and a highly effective and beneficial cleansing capacity with minimal skin interference and environmental impact.

## 2. Soaps vs. Syndets: Similarities and Differences

A diverse range of skin cleansers exist today, but they all generally fall into two types: soap-based and syndets. Both soap- and syndet-based cleansers contain at least one (often more than one) surfactant, a class of organic compounds that are amphiphilic/amphipathic, that is, they contain both nonpolar or hydrophobic (water-hating and lipid-loving) moieties, also known as ‘tails’, and polar or hydrophilic (water-loving) moieties, also known as ‘heads’. Therefore, they are soluble in both water and organic solvents [21]. Due to surfactants’ unique chemistry and characteristics, hydrophobic compounds present in excess sebum and other undesired substances (e.g., dirt, oils) on the skin’s surface are washed away with greater ease than could be achieved with water alone [7,9,12,13].

**Figure 1:**
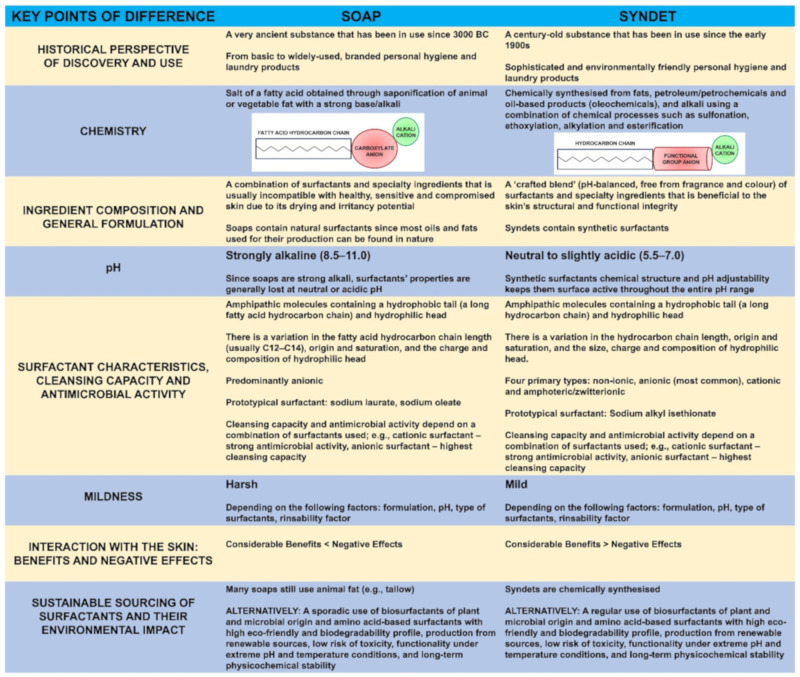
The key points of difference between soaps and syndets [3,9,10,13,16,17,19,20,21,22,23,24,25,26,27,28,29,30,31].

While soaps and syndets are similar in that they cleanse dirt and impurities from the surface of the skin, their distinct chemical properties and physiological effects can be markedly different [3,9,10,13,16,17,19,20,21,22,23,24,25,26,27,28,29,30,31] (Figure 1).

### 2.1. Soaps vs. Syndets: Key Points of Difference

The key points of difference between soaps and syndets (Figure 1) include the following: their historical perspective of discovery and use [19,29] (see Section 2.1.1); chemistry [9,16,24,29] (see Section 2.1.2); ingredient composition and general formulation [3,17,25] (see Section 2.1.3); pH (see Section 2.1.4) [22,24,25]; surfactant characteristics [13,21,32], cleansing capacity [13,16,21,27,32,33] and antimicrobial activity [23,28] (see Section 2.1.5); mildness [13,16,21,27] (see Section 2.1.6); sustainable sourcing of surfactants and their environmental impact [26,30,34,35,36] (see Section 2.1.7); and interaction with the skin [3], associated benefits and negative effects [3,9,10,20,25] (see Section 3).

#### 2.1.1. Historical Perspective of Discovery and Use

Traditional soap has existed as a cleanser for millennia, and as such, has a rich and long history (Figure 1). Early records show that soap-like materials were used by the Babylonians and Sumerians in 3000–2000 BC. Furthermore, there are references to soap in historical records from Ancient Egypt, Gaul and Classical Antiquity. For example, the inhabitants of Gaul, now Western Europe, used to prepare a specific substance from tallow (beef or sheep fat) and beech wood ash to dye their hair red and to treat a variety of skin conditions [29,37]. According to a Roman legend, the first ‘primitive’ soap making (what is today known as the process of saponification) [29,38] (see Section 2.1.2) took place on Mount Sapo (‘sapo’ meaning soap in Latin), a site close to Rome, where animals were sacrificed; animal fat was mixed with the plant ash and the addition of rainwater formed a mixture useful for washing clothing and skin [29]. In Europe, especially in the Mediterranean (Spain, France and Italy) soap making was well-established in the 9th and 10th century. In those days, soap was a luxury affordable only by the very rich. Mass production of soap started in the 19th century, and was a huge industry by the turn of the century, with multifarious soap bars available [37]. Still considered a medicinal product in the 19th century, it was not until the Act of 1975 in France, and then European Parliament and Council Directive 76/768/EEC10, that soap was categorised as a cosmetic. To comply with this new definition, there can now be no claim of any preventive or even curative property with regard to human conditions and diseases [29].

Syndets, on the other hand, are relative newcomers and have a much shorter, roughly 100 year history, with origins in the early 20th century (Figure 1). It was in the late 1940s following World War II that development of syndets took off to gradually gain major importance in the areas of personal hygiene and laundry products. Their relatively recent use corresponds to a societal need to have more environmentally friendly and milder-in-nature hygiene products that also come with minimal to no adverse effects [26,29].

#### 2.1.2. Chemistry

Soap is a salt of fatty acid (Figure 1) obtained through saponification (literally ‘conversion into soap’) of animal fat such as tallow and lard-pork fat (which contains fatty acids such as saturated palmitic and stearic acids and an unsaturated fatty acid such as oleic acid) or vegetable fat such as olive oil, palm oil and coconut oil with a strong base such as sodium hydroxide (caustic soda/lye), potassium hydroxide (potash) and magnesium hydroxide. The most common fatty acid chain lengths are usually in the C12–C18 range [29,30]. These combinations produce a number of different types of soap, including sodium tallowate, sodium palmate, sodium laurate, sodium cocoate, sodium oleate and magnesium stearate [9,24,29]. The choice between caustic soda and potash will influence the nature of the resulting soap at room temperature: soaps from caustic soda are solid (known as ‘white soaps’), whereas soaps from potash are liquid (known as ‘black soaps’) [29].

Syndets are chemically synthesised from fats, petroleum/petrochemicals, or oil-based products (oleochemicals) and alkali through a combination of chemical processes other than saponification, namely, sulfonation (the process of attaching the sulfonic acid, –SO_3_H functional group), ethoxylation (the process of attaching ethylene oxide, C_2_H_4_O) and esterification (carboxylic acids reacting with alcohols to form esters) [30] (Figure 1). Therefore, a syndet usually comprises a blend of synthetic compounds such as fatty acid isothionates or sulfosuccinic acid esters (e.g., alkyl sulfates and alkyl sulfosuccinates) [7,24,30].

#### 2.1.3. Ingredient Composition and General Formulation

Surfactants (be they non-ionic, anionic, cationic or amphoteric) (see Section 2.1.5) are the principal constituents of most soap and syndet formulations given that they are responsible for its cleansing action and antimicrobial activity [7]. The type, concentration and combination of surfactant, its rinsability factor and pH can have a bearing on the products resulting in drying and irritancy potential to healthy, compromised and diseased skin [7,39] (see Section 3).

In addition to surfactants, soaps and syndets also commonly contain a combination of some or most of the following ingredients: (1) water (or a suitable organic solvent) [7,39,40]; (2) barrier-maintaining and barrier-enhancing moisturisers to maintain and (re)hydrate the skin barrier [2,7,24,30,39]; (3) binders and plasticizers to stabilise the formulation [7,24,30,39]; (4) fillers to harden the formulation if required [24,30,39]; (5) lather/foam enhancers or boosters [24,39]; (6) preservatives to prevent the growth of microorganisms [24,30,39] and (7) fragrance and colour, which are usually only present in soaps [24,30,39].

#### 2.1.4. The Role of pH and Its Effect on the Skin’s Barrier Integrity

**Figure 2:**
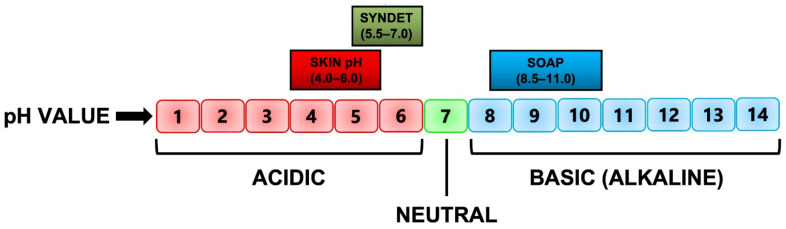
The pH range of the skin, syndet and soap, respectively [3,15,24,37,44,46].

As detailed above, the process of saponification requires the use of a strong base, which always results in a highly alkaline product. As the skin itself has a slightly acidic pH, between 4.0 and 6.0 [41,42,43,44,45,46] (Figure 2), application of an alkaline product to it will obviously lead more often than not to multiple negative effects. Conversely, syndets are usually the superior choice for all types of skin, as the processes used to create them allow for their pH to be adjusted to be similar to that of the skin, resulting in minimal disruption of the skin’s barrier and native microflora [24,30,39].

Soap has a pH typically in the range of 8.5–11.0, whereas the pH of a syndet is slightly acidic to neutral, with a range of 5.5–7.0, indicating an overlap between the pH of the skin and syndet and no overlap between the skin and soap [24] (Figure 2). Such differences in pH between soaps and syndets have an extensive effect on the interaction of skin cleansers with the SC [3,25] (see Section 3). For example, it has been reported that the skin proteins swell markedly if the cleanser pH is highly alkaline (pH > 8.0). Optical coherence tomography (OCT) images of the SC [9,37] after its exposure to acidic, neutral, and alkaline pH conditions, showed that there is significantly greater swelling of the SC in alkaline pH solutions. Alkaline pH also has an effect on SC lipids, as it can ionise fatty acids in the lipid bilayers, making them more ‘structurally like soap’ molecules that in turn can cause overall destabilisation of the lipid bilayers [37].

The pH of the skin is also essential for the adhesion of resident (e.g., *Staphylococcus epidermidis*) skin microflora. An acidic pH of the skin keeps the resident bacterial flora attached to the skin, whereas an alkaline pH promotes its dispersion from the skin. Swelling of the skin under alkaline conditions may potentially weaken the corneocytes, simultaneously allowing the resident bacteria to diffuse across the skin’s surface as well as diminishing their beneficial attributes, and permitting transient (opportunistic and pathogenic) bacteria such as *Staphylococcus aureus* to colonise the skin and thrive under such ‘heavenly’ conditions [23,41,47].

More recently, a new class of syndets that contains a combination of surfactants such as sulfosuccinate and acyl isethionate have pH values in the range of 5.0–5.5 [25,37]. Such syndets with pH being close to the skin’s native pH have ‘friendly’ mildness claims that imply less damaging effects to the skin. [3,15,37] (see Section 2.1.6 and Section 3).

#### 2.1.5. Surfactant Characteristics, Cleansing Capacity and Antimicrobial Activity

**Figure 3:**
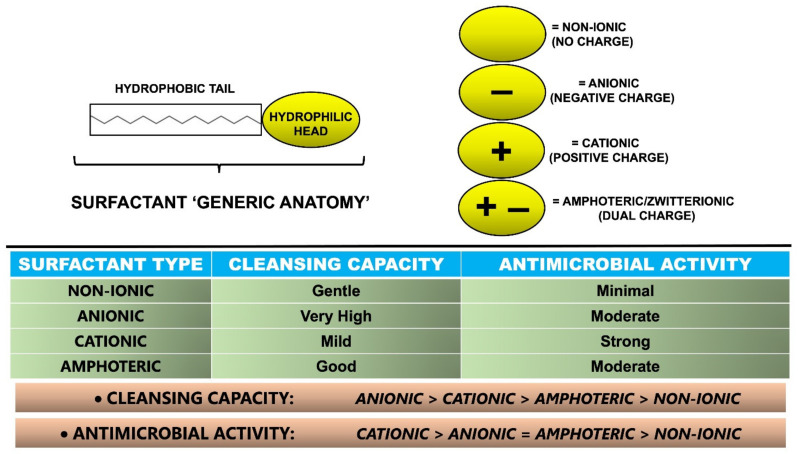
Categorisation of surfactants according to their hydrophilic head charge, and comparison of their associated cleansing capacity and antimicrobial activity [9,10,13,14,16,21,23,27,28,32,51].

Surfactants are broadly categorised into four major types based on the charge present in the hydrophilic head group (Figure 3): (1) non-ionic (no charge) (see Section Non-ionic Surfactants); (2) anionic (negative charge) (see Section Anionic Surfactants); (3) cationic (positive charge) (see Section Cationic Surfactants) and (4) amphoteric/zwitterionic (dual charge) (see Section Amphoteric/Zwitterionic Surfactants). Different types of surfactants are used individually or more commonly in combination with each other to integrate suitable properties (e.g., cleansing capacity, mildness, antimicrobial activity) (Figure 3) into different soap and syndet formulations (e.g., bar, wash, cream, lotion, gel) [7,14,21,32].

##### Non-Ionic Surfactants

Non-ionic surfactants (e.g., fatty alcohols and alcohol ethoxylates, polyoxyethylene family) have no electrical charge in their hydrophilic head. Their cleansing capacity (Figure 3) and lather characteristics are quite weak; therefore, they are considered the most gentle. Moreover, non-ionic surfactants usually have the lowest skin irritancy potential amongst the different types of surfactants [21,48,49]. Nevertheless, it has been reported [9,50] that non-ionic surfactants have a greater tendency to dissolve stearic acid than do anionic surfactants, which may translate into greater dissolution of skin lipids, if cleansers with excessive levels of non-ionic surfactants are used [9,21]. This hypothesis is consistent with transmission electron microscopic (TEM) studies [9,50] that show that non-ionic surfactant-based cleansers alter the lipid region to a greater extent than do mild cleansers with sodium cocoyl isethionate as the surfactant [51]. Among the compounds belonging to the group of non-ionic surfactants are alkyl polyglucosides, coco glucoside, lauryl glucoside and decyl glucoside [9,21].

There are a limited number of studies reporting on the antimicrobial activity of non-ionic surfactants. Nevertheless, it was found that C10E6 and C12E6 members of the polyoxyethylene family of surfactants showed efficacy against *Escherichia coli* (*E. coli*) [28]. The C10 surfactant adsorbed on the bacterial cell wall, whereas the C12 surfactant penetrated the membrane bilayer, and both pathways hindered bacterial cell viability. Other non-ionic surfactants such as C14 and C16 had no microbial effect [28].

##### Anionic Surfactants

Anionic surfactants are the most commonly used type of surfactant—together with cation surfactants, they are known as primary surfactants—in both soaps and syndets, constituting 70–75% of total worldwide surfactant consumption [14,21,32,52,53,54]. The members of this group have the highest cleansing capacity (Figure 3) and excellent lather characteristics, while generally being considered potent irritants to the skin [21]. Due to a negative charge and repelling action from most surfaces (which tend to be slightly negatively charged as well), anionic surfactants, in addition to their ability to emulsify oil and dirt into wash solutions, can lift contaminants and impurities from surfaces. Most anionic surfactants will generate significant foaming in solution, which is a desirable attribute in most cleansing applications. Anionic surfactants can be further subdivided according to their polar group chemistry into carboxylates, sulfates, sulfonate, phosphate esters and isethionates, with some specific examples including sodium laureth sulfate, sodium lauroyl sarcosinate, or sodium cocoyl isethionate. Furthermore, anionic surfactants are often combined with non-ionic and amphoteric surfactants, which are also known as secondary surfactants. Such addition of various non-ionic and amphoteric surfactants can decrease the irritancy potential of anionic surfactants and leave the skin with a pleasant feel [14,21,27,32,52,53,54].

Anionic surfactants have been leveraged as moderate antimicrobials (Figure 3). For example, it has been noted [28] that acidic, anionic formulas (containing ingredients structurally similar to the microbial cell wall lipids) at a pH of 2.0–3.0 aid in antimicrobial efficacy. The isoelectric points of many proteins (when the net charge of a protein becomes zero) are known to be within the pH range of 2.0–3.0, resulting in a switch in overall cell charge, from negative to positive [28].

##### Cationic Surfactants

Cationic surfactants represent one of the smaller classes of surfactants. These particular surfactants are positively charged and have lower and milder cleansing properties, but higher antimicrobial activity, than anionic surfactants (Figure 3). Cationic surfactants have irritancy potential similar to that of anionic surfactants, but may be marginally more damaging (when skin barrier damage and its permeability alterations become irreparable) to the skin [21]. The most common cationic surfactants are amine salts and quaternary ammonium salts (e.g., benzalkonium chloride). Benzalkonium chloride dissociates in aqueous solution to provide a relatively large and complex cation, which is responsible for the surface activity, and a smaller anion [28]. Additionally, it has broad-spectrum antimicrobial activity against an array of different microorganisms including bacteria (e.g., *S. aureus*), lipophilic viruses and fungi [55,56]. Benzalkonium chloride’s proposed antimicrobial mechanism of action involves five distinct steps: (1) adsorbs and penetrates the cell wall membrane; (2) disrupts lipid membranes to cause structural and functional disorder; (3) induces leakage and release of the cell’s content; (4) promotes breakdown of proteins, lipids and nucleic acids and (5) induces cell lysis, culminating in cell death [28,57].

##### Amphoteric/Zwitterionic Surfactants

Amphoteric surfactants exhibit the properties of either anionic or cationic surfactants [21,48]. They are generally less aggressive on the skin than anionic surfactants. Amphoteric surfactants are often used in combination with either anionic or cationic surfactants to enhance mildness [9,21]. Amphoteric surfactants also demonstrate good cleansing capacity (Figure 3) and lather characteristics, a moderate antimicrobial activity (Figure 3), compatibility with a wide pH range and a lack of cytotoxicity [21]. As such, these types of surfactants enjoy wide use. Commonly used amphoteric surfactants include cocamidopropyl betaine, lauryl betaine, sodium cocoamphoacetate and disodium cocoamphodiacetate [9,27,58].

Amphoteric surfactants have minimal impact on the biocidal activity of quaternary ammonium salts. Similarly to non-ionic surfactants, they are often used in antimicrobial preparations that contain cationic surfactants [58]. Furthermore, it was found that a synergy in antimicrobial performance exists between two quaternary ammonium salts, namely, cocoamidopropyl betaine and alkyl dimethyl amine oxide [28,59].

#### 2.1.6. The Concept of Mildness and Its Impact on the Skin

The ‘mildness’ of surfactants on the skin is of paramount importance due to their high frequency of use, especially when it comes to skin affected by conditions such as eczema and psoriasis. Thus, it is imperative to develop surfactants with as little impact as possible on the skin’s functional and structural integrity. Identification of the mechanisms underlying surfactant-related damage such as dryness or tightness through ultrastructural changes of the SC proteins and lipids should be very helpful in reducing the overall irritancy of surfactants [9,10,20,24,51,60]. There can be significant damage to both protein and lipid regions of the SC after multiple washes with a soap bar, whereas under the same conditions, skin cleansed with a syndet showed well-conserved protein and lipid regions of the SC [50]. This study also showed a good correlation between damage to SC ultrastructure and high TEWL [9,50]. The TEWL results, measured using an evaporimeter immediately after a wash, show that harsher soap induces a higher rate of evaporation than milder syndets [20]. Furthermore, a non-ionic surfactant-based cleanser resulted in a disrupted lipid region of the SC with minimal damage to proteins [9,50]. It was also shown that the soap-washed samples demonstrated significant uplifting of cells and surface roughness; by contrast, syndet-washed samples showed no signs of uplifting of cells. While these represent exaggerated conditions, they clearly demonstrate the potential for damage from soap systems (see Section 3). These results are consistent with the well-accepted mildness of syndets vs. soaps [9] (Figure 1).

#### 2.1.7. Sustainable Sourcing of Surfactants and Their Environmental Impact

Nowadays, a rise in product demand and environmental awareness is driving the development of a new generation of sustainable and environmentally friendly surfactants, including biosurfactants (green surfactants) of plant [36] (see Section Biosurfactants of Plant Origin) and microbial [35,36,61] (see Section Biosurfactants of Microbial Origin) origin and amino acid-based surfactants [34,62] (see Section Amino Acid-based Surfactants). When compared to conventional surfactants found in soaps and syndets, the main advantages of these novel surfactants include their high biodegradability profile, creating less hazardous waste, renewability, low risk of toxicity, functionality under extreme conditions (e.g., temperature and pH), and long-term physicochemical stability [35]. Such properties allow these surfactants to be used across various industries (e.g., food, cosmetic and pharmaceutical) in a range of processes (e.g., emulsification, separation) to reduce surface tension and to promote the penetration of bioactives through biological membranes [34,35,36,63].

Furthermore, unlike most conventional surfactants, the constituents of biosurfactants including sugars, lipids and proteins are similar to phospholipids, glycolipids and proteins found in the membrane of skin cells. The movement of molecules and compounds across the membrane of skin cells is dependent on their lipophilicity and surface activity; therefore, the unique structure of biosurfactants provides a high rate of permeability through the membrane of skin cells to regulate skin barrier functions. This subsequently triggers beneficial effects relating to skin protection mechanisms [64]. However, the application of biosurfactants as skin cleansers is still in its infancy.

##### Biosurfactants of Plant Origin

Plant-based surfactants are naturally omnipresent in different parts of plants, including the roots, stems, leaves, flowers, fruit, and seeds. Like any other conventional surfactants, they are amphiphatic molecules that constitute a diverse group of compounds characterised by a structure made up of phospholipids (e.g., lecithin, phosphatidylcholine, phosphatidylethanolamine, phosphatidylinositol), proteins or protein hydrolysates [36] and saponins [36,65]. Recently, it was reported that lecithin can be useful as a skin-friendly surfactant for application in skin care products [66]. Its impact on skin barrier function was found to be considerably lower than that of strong, conventional anionic surfactants such as harsh sodium lauryl sulfate/sodium dodecyl sulfate. Thus, its use on sensitive skin with pre-existing conditions such as eczema can be considered and should be explored further [66].

##### Biosurfactants of Microbial Origin

Biosurfactants of microbial origin are a structurally diverse group of compounds that includes phospholipids, fatty acids, glycolipids, lipopeptides (e.g., surfactin) and lipopolysaccharides. The hydrophilic portion of a microbial biosurfactant can be composed of a carbohydrate, amino acid, cyclic peptide, phosphate, carboxylic acid, or alcohol, while the hydrophobic portion can be composed of long-chain fatty acids or hydroxylated fatty acids. A variety of microorganisms, including bacteria (e.g., *Bacillus subtilis*, *Pseudomonas aeruginosa*), yeasts (e.g., *Candida lipolytica*, *Candida bombicola*) and filamentous fungi (e.g., *Phoma herbarum*), can effectively produce biosurfactants with different molecular structures and surface activities [35,36,67].

For instance, surfactin produced by the bacterium *B. subtilis* is one of the most active surface biosurfactants that possesses an expressive surface activity and interfacial tension under harsh physical and chemical conditions. Surfactin is able to reduce the surface tension of water from 72 mN/m to 27 mN/m (milliNewtons/meter) [35]. The antimicrobial activities of surfactin biosurfactants are believed to be due to their accumulation on the bacterial cell membrane surfaces until a threshold concentration is reached, enabling their penetration into the membrane to cause further cellular disintegration [64]. As such, the antimicrobial potential of microbial surfactin biosurfactants against pathogenic skin bacteria and microorganisms in general remains promising.

##### Amino Acid-Based Surfactants

Amino acid-based surfactants are synthetic equivalents to biosurfactants. As novel surfactants, they can be synthesised via different biotechnological and chemical routes using renewable sources (i.e., amino acids). Their high degradability and harmless by-products make them safer for our environment [34]. There are a total of 20 standard amino acids that are responsible for cell growth and all physiological reactions in living organisms. Some amino acids are hydrophobic, others are hydrophilic, some are basic and some are acidic, making it possible to produce an extensive range of surfactants with great potential as biocompatible, sustainable and eco-friendly substances. Some examples include non-ionic phenylalanine- and leucine-derived surfactants; cationic cocoyl arginine ethyl ester; anionic sodium *N*-cocoyl-l-glutamate and potassium *N*-cocoyl glycinate; amphoteric lauroyl lysine and alkoxy (2-hydroxypropyl) arginine [34,62]. Their simple and natural structures, low toxicity, mildness and rapid biodegradation often make them superior to their traditional counterparts. For example, potassium *N*-cocoyl glycinate was found to be mild to skin when applied and used in face cleansers. Similarly, *N*-dodecanoylalaninate is known to produce a non-irritating creamy foam, making it particularly suitable for products intended for the likes of baby skin [34].

## 3. Effects of Mild Syndets and Harsh Soaps on the Skin

**Figure 4:**
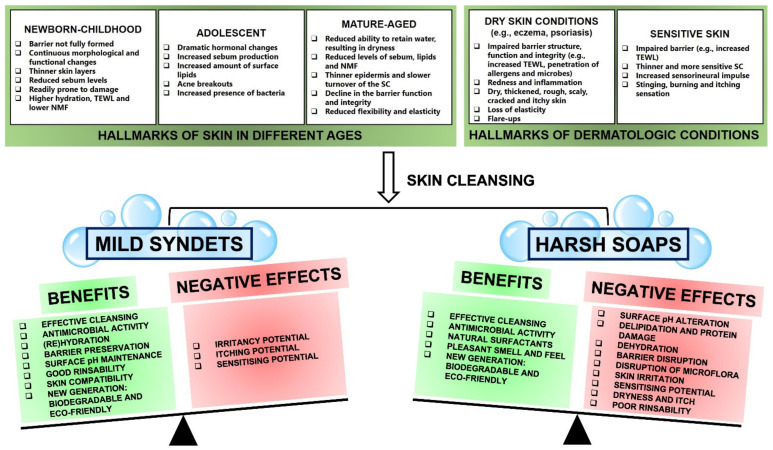
Benefits and negative effects of mild syndets and harsh soaps exhibited on the skin in different ages and common dermatologic conditions [7,9,12,13,23,41].

Selecting an optimal and suitable cleanser is key to maintaining the skin acid mantle, its barrier function and integrity, and preserving the skin’s overall health at the same time. The exact needs of the skin differ with age and skin condition (Figure 4). Therefore, it is essential to understand these needs and differences in order to identify the most suitable cleanser for every situation. For instance, thinner skin can be more prone to TEWL, so soaps may increase this negative effect by exacerbating TEWL, whereas syndets may limit further TEWL and improve hydration. It seems that the interaction of mild syndets with the skin can be beneficial for almost every situation (see Section 3.1) and will tip the balance as the positives outweigh the negatives, whereas the interaction of harsh soaps with the skin can have a much greater potential to cause a range of negative effects such as impaired barrier function, dryness and irritancy, and will tip the balance towards the negative effects (see Section 3.2) (Figure 4). Therefore, fine-tuning of the complex characteristics of cleansers is absolutely necessary in order to produce cleansing formulations with optimal benefits and minimal adverse effects [7,9,12,13].

### 3.1. Mild Syndets: Considerable Benefits and Minimal Negative Effects

Children in general, especially newborns and infants, have a unique skin structure and physiology. While newborn skin is still not fully formed, it is sufficiently mature to cope with the usual demands of life. The skin continuously undergoes a period of rapid anatomical and physiological transformation, particularly in relation to SC hydration, TEWL, sebum and NMF production, development of the skin barrier (in terms of structural and functional integrity), the transition to a more acidic skin surface and skin maturation [13,68,69]. This process of maturation and morphological and functional changes continues for several years, indicating that newborn-childhood skin is particularly vulnerable to sensitisation, inflammation, irritation and unfavourable changes in skin barrier function [13,69] (Figure 4). Therefore, it is an imperative to use a suitable cleanser with mild properties for a child’s skin [69].

At puberty, the levels of circulating growth and reproduction hormones increase dramatically, causing various changes in the skin, including a high prevalence of acne [13]. A defining characteristic of acne is abnormal sebum production, making the skin oily, an issue that can be compounded by the drying nature of acne medication, such as benzoyl peroxide. Thus, effective cleansers, especially facial cleansers for acne management, must satisfy two opposing needs: (1) removal of sebum and (2) maintaining skin moisture [13]. A mild, fragrance-free and irritant-free cleanser with good rinsability is the recommended cleanser for acne management [7,13]. The cleansing regimen should suit the needs of the individual patient [7,14] (Figure 4).

At the other end of the spectrum, aged skin is characterised by changes such as impaired barrier function, thinner epidermis, reduced skin elasticity and decreased sweat and sebum production. Such changes ultimately result in a reduced ability of the skin to retain water, eventually leading to dry and fragile skin [13,70]. When it comes to cleansing aged skin, the recommendations remain the same as that for young skin: avoid alkaline cleansers and use products that are mild in nature, and able to maintain or even replenish the skin’s moisture [70] (Figure 4).

In individuals with dry skin conditions, including eczema, the use of traditional soap with its characteristic high pH can aggravate the skin, leading to loss of intracellular lipids, leaving the skin with a red, rough and scaly appearance [13,71]. This damage can potentially expose dermal nerve endings (a hallmark of sensitive skin), resulting in itching, burning, and pain [72]. Skin barrier impairment can also contribute to the penetration of allergens and an increased colonisation of bacteria such as *S. aureus*. Again, when it comes to cleansing, the recommendations remain similar: use mildly acidified syndets for cleansing, with an adjusted pH value in order to reduce the participation of infectious organisms, irritants, or allergens as well as to minimise irritation and itching potential [7,13,14,71] (Figure 4).

Unfortunately, even syndets with favourable mildness can potentially remove skin’s essential constituents, compromise the integrity and functionality of the SC, and can inevitably result in some weakening of the skin barrier, sensitisation and irritation (Figure 4). Thus, skin cleansing activity should be conducted with care because the careless use of skin cleansers in general, and especially the use of harsh alkaline soaps, will undoubtedly cause adverse skin reactions [73]. A best-suited and mild-in-nature cleanser such as a syndet should effortlessly and simultaneously maintain a fine balance between skin cleansing on the one hand and the preservation of the skin’s homeostatic properties on the other, with minimal to no irritation, disruption of, or damage to the skin’s physiological parameters, including hydration, acid mantle, and thus, overall barrier function [3,7].

### 3.2. Harsh Soaps: Extensive Short-Term and Long-Term Negative Effects and Some Benefits

**Figure 5:**
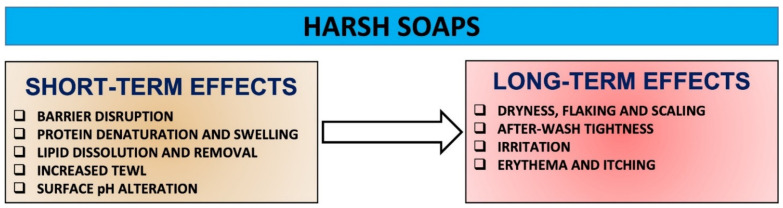
The nexus between the use of harsh soaps and their negative impact on the skin over time [7,14,20].

Harsh soaps have the potential and aggressiveness to initiate changes to SC proteins, lipids and pH, disrupt resident microflora, and progressively increase damage over time, which in turn can cause a fundamental destruction of the barrier integrity that underpins skin health (Figure 4). Overall, the use of harsh soaps can cause cumulative skin effects including dryness, roughness, scaling, after-wash tightness, irritation and itching [7,14,17,20,41] (Figure 5).

Most of the water absorbed by the SC during cleansing is present within the corneocytes. This results in a significant protein denaturation and swelling, and ultimate keratinocyte damage. Harsh soaps increase the swelling further, and the extent of surfactant-induced swelling is dependent upon the surfactant’s nature and its irritation potential. In addition, harsh soaps binding to proteins may also reduce the water-holding capacity of proteins. For example, the extent of swelling in the presence of sodium laurate-containing soap is significantly higher than that in the presence of a sodium cocoyl isethionate-containing syndet [20]. Furthermore, harsh soaps have been shown to remove NMF components and cause damage to the corneocyte envelope [20]. Therefore, the higher loss of water-soluble proteins after a single wash with soap vs. syndet is consistent with the greater damage potential of soap, depending on its structural and charge-density differences, direct effects of pH on the SC, and/or indirect effects of pH on the solution chemistry of charged head groups [7,14,20]. As water evaporates at a rapid rate from the upper layers of the skin, a differential stress is created in the SC, and this is thought to be the origin of the after-wash tightness [20].

Harsh soaps have an ability to disrupt and potentially damage bilayer lipids by extracting endogenous cholesterol, fatty acids and ceramides or intercalating into the lipid bilayer. Lipid barrier dissolution has numerous clinical consequences for the skin, including dryness, increased TEWL, fissuring, flaking, erythema and itch [14]. For example, insertion of anionic surfactants into the lipid bilayer can induce charge disruption in the bilayer and abruptly disturb membrane packing and organisation, permeability and overall structural and functional integrity. Even a subtle or partial removal of such lipids can make the lipid bilayer unstable [14,20]. It was shown that a harsh soap removes more cholesterol than a syndet [20]. While the exact reasons for this difference are not clear at present, it is likely that the alkaline pH of soap allows ionisation of the bilayer fatty acids, allowing easier cholesterol extraction from the SC. Furthermore, it is possible that the increased swelling of soap-damaged SC allows deeper layers of the SC to be readily exposed to the soap [20].

## 4. Conclusions

The main objective of skin hygiene is to maintain the skin’s cleanliness, freshness, radiance and overall health but without removing protective SC surface proteins and lipids, affecting skin microbiota or changing pH. Surfactants are the workhorse ingredients of soaps and syndets responsible for skin cleansing, and thus, overall skin health. Soaps and syndets are divergent and very complex molecules whose interactions with the skin are driven by several factors regarding the surfactants’ presence, composition, origin and diversity, pH, skin compatibility, cleansing capacity and effectiveness, rinsability, and mildness/harshness. Given the incompatibility of traditional soap with the skin, mild syndets are the preferred choice for providing cleansing, antimicrobial protection and overall skin hygiene to healthy, compromised and diseased skin alike. Harsh soaps readily extract endogenous skin components such as proteins and lipids during cleansing and remain on the skin’s surface after rinsing. Such negative effects disrupt skin structural and functional integrity and degrade its barrier properties over time. Therefore, it is absolutely necessary to understand the physicochemical properties of skin cleansers, their biological activity, use and potential side effects in order to successfully optimise existing formulations or develop novel skin cleansers (even personalised skin cleansers) that can benefit both the skin and the environment with minimal consequences.

## Data Availability

Not applicable.

## References

[1] Évora A.S., Adams M.J., Johnson S.A., Zhang Z. (2021). Corneocytes: Relationship between Structural and Biomechanical Properties. Ski. Pharmacol. Physiol..

[2] Del Rosso J.Q., Levin J. (2011). The clinical relevance of maintaining the functional integrity of the stratum corneum in both healthy and disease-affected skin. J. Clin. Aesthetic Dermatol..

[3] Mukherjee S., Chandrashekar B., Gaikwad R. (2015). Cleansers: To use or not to use. Indian J. Paediatr. Dermatol..

[4] Das C., Olmsted P.D. (2016). The physics of stratum corneum lipid membranes. Philos. Trans. R. Soc. London. Ser. A Math. Phys. Eng. Sci..

[5] Lefèvre-Utile A., Braun C., Haftek M., Aubin F. (2021). Five Functional Aspects of the Epidermal Barrier. Int. J. Mol. Sci..

[6] Dingjan T., Futerman A.H. (2021). The fine-tuning of cell membrane lipid bilayers accentuates their compositional complexity. BioEssays.

[7] Mukhopadhyay, P. (2011). Cleansers and their role in various dermatological disorders. Indian J. Dermatol..

[8] Mundstock A., Abdayem R., Pirot F., Haftek M. (2014). Alteration of the Structure of Human Stratum Corneum Facilitates Transdermal Delivery. Open Dermatol. J..

[9] Ananthapadmanabhan K.P., Moore D., Subramanyan K., Misra M., Meyer F. (2004). Cleansing without compromise: The impact of cleansers on the skin barrier and the technology of mild cleansing. Dermatol. Ther..

[10] Ananthapadmanabhan K., Mukherjee S., Chandar P. (2013). Stratum corneum fatty acids: Their critical role in preserving barrier integrity during cleansing. Int. J. Cosmet. Sci..

[11] Wilson M. (2017). The importance of skin cleansing for people with incontinence. Nurs. Resid. Care.

[12] Draelos Z., Hornby S., Walters R., Appa Y. (2013). Hydrophobically modified polymers can minimize skin irritation potential caused by surfactant-based cleansers. J. Cosmet. Dermatol..

[13] Reis C.M.S., Reis-Filho E. (2016). Cleansers.

[14] Navare B., Thakur S., Nakle S. (2019). A review on surfactants: Role in skin, irritation, SC damage, and effect of mild cleansing over damaged skin. Int. J. Adv. Res. Ideas Innov. Technol..

[15] Greive K. (2015). Cleansers and moisturisers: The basics. Wound Pract. Res. J. Aust. Wound Manag. Assoc..

[16] Li Z. (2020). Modern Mild Skin Cleansing. J. Cosmet. Dermatol. Sci. Appl..

[17] Walters R.M., Mao G., Gunn E.T., Hornby S. (2012). Cleansing Formulations That Respect Skin Barrier Integrity. Dermatol. Res. Pr..

[18] Wong R., Geyer S., Weninger W., Guimberteau J.-C., Wong J.K. (2016). The dynamic anatomy and patterning of skin. Exp. Dermatol..

[19] Routh H.B., Bhowmik K.R., Parish L.C., Witkowski J.A. (1996). Soaps: From the phoenicians to the 20th century—A historical review. Clin. Dermatol..

[20] Flynn T.C., Petros J., Clark R.E., Viehman G.E. (2001). Dry skin and moisturizers. Clin. Dermatol..

[21] Corazza M., Lauriola M.M., Zappaterra M., Bianchi A., Virgili A. (2010). Surfactants, skin cleansing protagonists. J. Eur. Acad. Dermatol. Venereol..

[22] Correa M.C.M., Nebus J. (2012). Management of Patients with Atopic Dermatitis: The Role of Emollient Therapy. Dermatol. Res. Pract..

[23] Duncan C.N., Riley T.V., Carson K.C., Budgeon C.A., Siffleet J. (2013). The effect of an acidic cleanser versus soap on the skin pH and micro-flora of adult patients: A non-randomised two group crossover study in an intensive care unit. Intensiv. Crit. Care Nurs..

[24] Friedman M., Spitz L. (2016). Chemistry, Formulation, and Performance of Syndet and Combo Bars. Soap Manufacturing Technology.

[25] Draelos Z.D. (2018). The science behind skin care: Cleansers. J. Cosmet. Dermatol..

[26] Kogawa A.C., Cernic B.G., Couto L.G.D.D., Salgado H.R.N. (2017). Synthetic detergents: 100 years of history. Saudi Pharm. J..

[27] Cornwell P.A. (2018). A review of shampoo surfactant technology: Consumer benefits, raw materials and recent developments. Int. J. Cosmet. Sci..

[28] Falk N.A. (2019). Surfactants as Antimicrobials: A Brief Overview of Microbial Interfacial Chemistry and Surfactant Antimicrobial Activity. J. Surfactants Deterg..

[29] Coiffard L., Couteau C. (2020). Soap and syndets: Differences and analogies, sources of great confusion. Eur Rev. Med. Pharm. Sci.

[30] Chirani M.R., Kowsari E., Teymourian T., Ramakrishna S. (2021). Environmental impact of increased soap consumption during COVID-19 pandemic: Biodegradable soap production and sustainable packaging. Sci. Total Environ..

[31] Oyekunle J.A., Ore O.T., Ogunjumelo O.H., Akanni M.S. (2021). Comparative chemical analysis of Indigenous Nigerian soaps with conventional ones. Heliyon.

[32] Belhaj A.F., Elraies K.A., Mahmood S.M., Zulkifli N.N., Akbari S., Hussien O.S. (2019). The effect of surfactant concentration, salinity, temperature, and pH on surfactant adsorption for chemical enhanced oil recovery: A review. J. Pet. Explor. Prod. Technol..

[33] Nazdrajic S., Bratovcic A. (2019). The role of surfactants in liquid soaps and its antimicrobial properties. Int. J. Adv. Res..

[34] Tripathy D.B., Mishra A., Clark J., Farmer T. (2018). Synthesis, chemistry, physicochemical properties and industrial applications of amino acid surfactants: A review. Comptes Rendus. Chim..

[35] Bjerk T.R., Severino P., Jain S., Marques C., Silva A.M., Pashirova T., Souto E.B. (2021). Biosurfactants: Properties and Applications in Drug Delivery, Biotechnology and Ecotoxicology. Bioeng..

[36] Farias C.B.B., Almeida F.C., Silva I.A., Souza T.C., Meira H.M., Silva R.D.C.F.S.D., Luna J.M., Santos V.A., Converti A., Banat I.M. (2021). Production of green surfactants: Market prospects. Electron. J. Biotechnol..

[37] Johnson A.W., Ananthapadmanabhan K., Hawkins S., Nole G., Draelos Z. (2016). Bar Cleansers. Cosmetic Dermatology.

[38] Rahayu S., Pambudi K.A., Afifah A., Fitriani S.R., Tasyari S., Zaki M., Djamahar R. (2021). Environmentally safe technology with the conversion of used cooking oil into soap. J. Phys. Conf. Ser..

[39] Kuehl B.L., Fyfe K.S., Shear N.H. (2003). Cutaneous cleansers. Ski. Ther. Lett..

[40] Pohorille A., Pratt L.R. (2012). Is Water the Universal Solvent for Life?. Orig. Life Evol. Biosph..

[41] Lambers H., Piessens S., Bloem A., Pronk H., Finkel P. (2006). Natural skin surface pH is on average below 5, which is beneficial for its resident flora. Int. J. Cosmet. Sci..

[42] Schmid-Wendtner M.-H., Korting H.C. (2006). The pH of the Skin Surface and Its Impact on the Barrier Function. Ski. Pharmacol. Physiol..

[43] Ali S.M., Yosipovitch G. (2013). Skin pH: From Basic SciencE to Basic Skin Care. Acta Derm. Venereol..

[44] Wohlrab J., Gebert A. (2018). pH and Buffer Capacity of Topical Formulations. Curr. Probl. Dermatol..

[45] Lukić M., Pantelić I., Savić S. (2021). Towards Optimal pH of the Skin and Topical Formulations: From the Current State of the Art to Tailored Products. Cosmetics.

[46] Brinke A.S.T., Mehlich A., Doberenz C., Janssens-Böcker C. (2021). Acidification of the Skin and Maintenance of the Physiological Skin pH Value by Buffered Skin Care Products Formulated around pH 4. J. Cosmet. Dermatol. Sci. Appl..

[47] Diaz D., Ditre C.M. (2020). The Effect of Cleansers on the Skin Microbiome. Pract. Dermatol..

[48] Effendy I., Maibach H.I. (1995). Surfactants and experimental irritant contact dermatitis. Contact Dermat..

[49] Hepworth P., Farn. R.J. (2006). Non-ionic surfactants. Chemistry and Technology of Surfactants.

[50] Misra M., Ananthapadmanabhan K.P., Hoyberg K., Gursky R.P., Prowell S., Aronson M.P. (1997). Correlation between sur-fac-tant-induced ultrastructural changes in epidermis and transepidermal water loss. J. Soc. Cosmet. Chem..

[51] Ananthapadmanabhan K.P., Lang Y., Vincent C., Tsaur L., Vetro K., Foy V., Zhang S., Ashkenazi A., Pashkovski E., Subramanian V. (2009). A novel technology in mild and moisturising cleansing liquids. Cosmet. Dermatol..

[52] Hibbs J., Farn. R.J. (2006). Anionic surfactants. Chemistry and Technology of Surfactants.

[53] Myers D. (2006). Surfactant Science and Technology.

[54] Dave N., Joshi T. (2017). A concise review on surfactants and its significance. Int. J. Appl. Chem..

[55] Kim M., Weigand M.R., Oh S., Hatt J.K., Krishnan R., Tezel U., Pavlostathis S.G., Konstantinidis K.T. (2018). Widely Used Benzalkonium Chloride Disinfectants Can Promote Antibiotic Resistance. Appl. Environ. Microbiol..

[56] Merchel Piovesan Pereira B., Tagkopoulos I. (2019). Benzalkonium Chlorides: Uses, Regulatory Status, and Microbial Resistance. Appl. Environ. Microbiol..

[57] Gilbert P., Moore L.E. (2005). Cationic antiseptics: Diversity of action under a common epithet. J. Appl. Microbiol..

[58] Otterson R., Farn. R.J. (2006). Amphoteric surfactants. Chemistry and Technology of Surfactants.

[59] Krasowska A., Biegalska A., Lukaszewicz M. (2012). Comparison of antimicrobial activity of three commercially used quaternary ammonium surfactants. Sepsis.

[60] Ananthapadmanabhan K.P. (2019). Amino-Acid Surfactants in Personal Cleansing (Review). Tenside Surfactants Deterg..

[61] Moldes A., Rodríguez-López L., Rincón-Fontán M., López-Prieto A., Vecino X., Cruz J. (2021). Synthetic and Bio-Derived Surfactants Versus Microbial Biosurfactants in the Cosmetic Industry: An Overview. Int. J. Mol. Sci..

[62] Infante M.R., Perez L., Pinazo A., Clapes P., Moran M.D.C., Angelet M., Garcia M.T., Vinardell M.P. (2004). Amino acid-based surfactants. Comptes Rendus. Chim..

[63] Singh P., Patil Y., Rale V. (2018). Biosurfactant production: Emerging trends and promising strategies. J. Appl. Microbiol..

[64] Adu S.A., Naughton P.J., Marchant R., Banat I.M. (2020). Microbial Biosurfactants in Cosmetic and Personal Skincare Pharmaceutical Formulations. Pharmaceutics.

[65] Rai S., Acharya-Siwakoti E., Kafle A., Devkota H.P., Bhattarai A. (2021). Plant-Derived Saponins: A Review of Their Surfactant Properties and Applications. Science.

[66] Vater C., Apanovic A., Riethmüller C., Litschauer B., Wolzt M., Valenta C., Klang V. (2021). Changes in Skin Barrier Function after Repeated Exposition to Phospholipid-Based Surfactants and Sodium Dodecyl Sulfate In Vivo and Corneocyte Surface Analysis by Atomic Force Microscopy. Pharmaceutics.

[67] Da Silva A.F., Banat I.M., Giachini A.J., Robl D. (2021). Fungal biosurfactants, from nature to biotechnological product: Bioprospection, production and potential applications. Bioprocess. Biosyst. Eng..

[68] Stamatas G.N., Walters R.M., Martin K.M. (2011). Formulating for unique needs of baby skin. Personal Care.

[69] Hugill K. (2014). Neonatal skin cleansing revisited: Whether or not to use skin cleansing products. Br. J. Midwifery.

[70] Farage M.A., Miller K.W., Elsner P., Maibach H.I. (2007). Structural Characteristics of the Aging Skin: A Review. Cutan. Ocul. Toxicol..

[71] Solodkin G., Chaudhar U., Subramanyan K., Johnson A.W., Yan X., Gottlieb A. (2006). Benefits of mild cleansing: Synthetic sur-factant based (syndet) bars for patients with atopic dermatitis. Cutis.

[72] Duarte I., Silveira J.E.P.S., Hafner M.D.F.S., Toyota R., Pedroso D.M.M. (2017). Sensitive skin: Review of an ascending concept. An. Bras. Dermatol..

[73] Surber C., Dragicevic N., Kottner J. (2018). Skin Care Products for Healthy and Diseased Skin. Metab. Disord. Nutr. Correl. Ski..

